# Five million nights: temporal dynamics in human sleep phenotypes

**DOI:** 10.1038/s41746-024-01125-5

**Published:** 2024-06-20

**Authors:** Varun K. Viswanath, Wendy Hartogenesis, Stephan Dilchert, Leena Pandya, Frederick M. Hecht, Ashley E. Mason, Edward J. Wang, Benjamin L. Smarr

**Affiliations:** 1 Department of Electrical and Computer Engineering, Jacobs School of Engineering, University of California, San Diego, CA USA; 2 Osher Center for Integrative Health, University of California, San Francisco, CA USA; 3 Zicklin School of Business, Baruch College, The City University of New York US, New York, NY USA; 4 Shu Chien—Gene Lay Department of Bioengineering, Jacobs School of Engineering, University of California, San Diego, CA USA; 5 Halıcıoğlu Data Science Institute, University of California, San Diego, CA USA

**Keywords:** Statistical methods, Computational models

## Abstract

Sleep monitoring has become widespread with the rise of affordable wearable devices. However, converting sleep data into actionable change remains challenging as diverse factors can cause combinations of sleep parameters to differ both between people and within people over time. Researchers have attempted to combine sleep parameters to improve detecting similarities between nights of sleep. The cluster of similar combinations of sleep parameters from a night of sleep defines that night’s sleep phenotype. To date, quantitative models of sleep phenotype made from data collected from large populations have used cross-sectional data, which preclude longitudinal analyses that could better quantify differences within individuals over time. In analyses reported here, we used five million nights of wearable sleep data to test (a) whether an individual’s sleep phenotype changes over time and (b) whether these changes elucidate new information about acute periods of illness (e.g., flu, fever, COVID-19). We found evidence for 13 sleep phenotypes associated with sleep quality and that individuals transition between these phenotypes over time. Patterns of transitions significantly differ (i) between individuals (with vs. without a chronic health condition; chi-square test; *p*-value < 1e−100) and (ii) within individuals over time (before vs. during an acute condition; Chi-Square test; *p*-value < 1e−100). Finally, we found that the patterns of transitions carried more information about chronic and acute health conditions than did phenotype membership alone (longitudinal analyses yielded 2–10× as much information as cross-sectional analyses). These results support the use of temporal dynamics in the future development of longitudinal sleep analyses.

## Introduction

Sleep monitoring has become widespread with the rise of affordable wearable devices^1^. While the National Institutes of Health (NIH) recommends that adults obtain 7–9-h of sleep in the form of a single (monophasic) sleep period per 24-h period, large-scale, real-world sleep studies observed a diversity of sleep structures, from short monophasic to varied-length polyphasic, and sleep characteristics^2–5^. Researchers have attributed such variability, both between people and within people over time, to several health- and daily living-related factors^2–9^. Because of this variability, converting sleep data from individuals in the real world into actionable insights to improve one’s health remains challenging. To make it possible to develop tools that provide such health insights, we need better methods for quantifying differences between people and within people over time.

Researchers have developed a promising large-scale sleep data clustering model to identify clusters of sleep with insomnia-like characteristics^2^. Katori et al. captured characteristics of an individual’s sleep behavior using sleep features (e.g., sleep time, sleep percentage, and number of sleep segments) extracted from 7-night periods of sleep. They extracted several clusters from a topological manifold of the sleep features and referred to these clusters as sleep phenotypes. They identified “insomnia-like” phenotypes characterized by clusters of nights with short, highly fractured sleep, as well as phenotypes related to social jet lag and shift work. These results suggest that sleep phenotypes derived from a topological manifold could be a useful classification to quantify differences in sleep behavior between people.

However, such large-scale analyses of sleep phenotype have, to date, used cross-sectional data, which preclude longitudinal analyses that could better quantify differences within individuals. Individual differences in sleep are relevant to acute conditions, such as illness, as well as changes in behavior, such as those related to consumption, exercise, or stress, and thus are essential for sleep-based assessment of one’s health. If sleep phenotypes represent meaningful differences in sleep behavior, then within-individual transitions between phenotypes over time in a population, which we refer to as temporal dynamics, should be related to the population’s health. Thus, we hypothesized that the temporal dynamics of a healthy population, primarily driven by daily living, should be different from those of an ill population.

Here, we assess whether an individual’s position in a clustering model of sleep phenotypes changes over time and whether such changes contain additive information about health conditions. Our dataset of 5.10 million nights over 33,152 individuals allows us to investigate this question in greater depth and at higher resolution compared with previous studies, which were based on only 3–7 recorded sleep nights per participant (Katori et al.). We adapt Katori et al.’s data processing pipeline to our 5-million-night dataset while treating each of our sleep periods (3-6 nights) independently and obtain a set of cohesive sleep phenotypes that include both a recommended sleep phenotype (8-hour and monophasic) and insomnia-like sleep phenotypes (<6.5 h and/or segmented). We discover a spectrum of variability in the degree to which individuals remain in the same cluster over time. We model possible movements from one cluster to another as edges between nodes in a graph and set the weight of each edge as the probability that an individual in a given population transitions between the clusters associated with that edge. To assess relevance to particular health conditions, we test (i) whether patterns in transition probabilities of a chronically ill cohort differ from those of a generally healthy cohort and (ii) whether patterns in transition probabilities during illness differ from before illness in the same group of individuals. We discover that these temporal dynamics are perturbed by various health conditions, including diabetes, sleep apnea, flu, fever, and COVID-19. We further find that our temporal dynamics model captures multiplicatively more information about these health conditions than static clusters on its own. Our findings show that sleep phenotypes change over time and that temporal sleep dynamics carry significant, nonrandom information about a range of health conditions.

## Results

### Adaptation of previous sleep data clustering model to TemPredict Dataset

We performed our analyses using sleep-wake time series and self-report survey responses collected from 33,152 individuals (age = 44.4 ± 12 years (mean ± std.); 19,792 males; 12,571 females; 32 others; 757 unknown; see Table 1 for more detail) between January 1st and October 24th of 2020, as part of the TemPredict Study^10^. We obtained usable, wearable data from these participants using a commercially available smart-ring wearable device (Oura Ring) for 4,682,978 (91.89%) nights out of 5,095,798 potential nights of data.

**Table 1:** Demographics of participants (*N* = 33,152; 757 unknown)

	Age bin							
Sex	18–19	20–29	30–39	40–49	50–59	60–69	70–79	80+
Female	51	1145	2952	3738	2965	1349	349	22
Male	79	2271	5539	5930	3819	1628	494	32
Other	–	2	7	9	8	4	1	1

Number of participants in each demographic by age and sex.

**Fig. 1:**
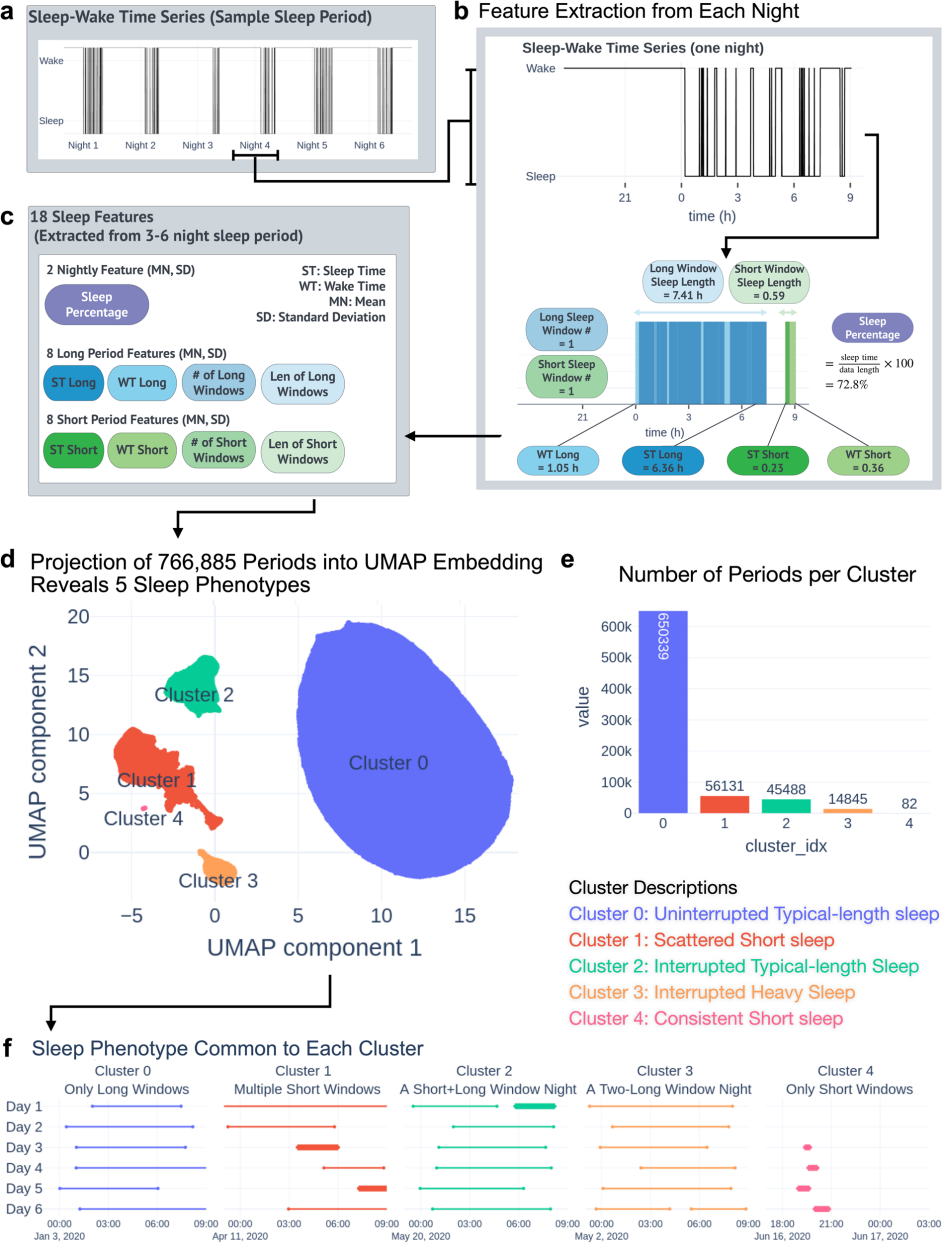
Obtaining Sleep Phenotypes from Five Million Nights of Sleep. Construction of sleep landscape and high-level phenotypes. **a**, **b** 3–6-night sleep periods’ timing (top left) are broken down by night (top right), and assessed for duration of individual sleep and wake bouts. **c** Each night is featured into numerical values for each of the 18 parameters (bottom; blue: long window parameters; green: short window parameters). **d** Projection of features into 2D space defined by first two UMAP components; 5 clusters are revealed. **e** Total number of 3–6-night sleep periods in each cluster. **f** Example of a typical 3–6-night sleep period from each cluster with cluster characteristics used to describe sleep phenotypes. Short windows are shown with thick lines, while long windows are shown with thin lines.

As shown in Fig. 1a, b, we first deconstructed each night of a sleep–wake time series into sets of long and short sleep periods that represent sleep structure and then extracted sleep quality features from each type of sleep period as defined in prior work^2^. As shown in the bottom half of Fig. 1b, we extracted nine sleep characteristics to capture the architecture of an individual’s sleep in each night. We applied similar steps as Katori et al. for constructing our long and short windows from the sleep–wake time series and reported the mean and standard deviation of each index shown in Table 2. Katori et al. found a Sleep Time Long mean of 6.60 hours while we found Sleep Time Long to be 6.99 h on average. Katori et al. found a Wake Time Long mean of 170.25 min, while we found a Wake Time Long of 64.4 min on average. We expected differences in these sleep feature values for two reasons: Our population was 44.4 years old on average compared with 62 years for the population studied by Katori et al. In addition, our population was self-selected from Oura Ring users, while the Katori et al. population represents a “healthy volunteer” selection bias^11^; for both reasons, some differences in sleep feature values are to be expected.

**Table 2:** Statistics of nine nightly sleep feature values

	Sleep features								
Stat.	Sleep percent (%)	ST long (h)	WT long (h)	ST short (h)	WT short (h)	LW count (#)	SW count (#)	LW length (h)	SW length (h)
Mean	0.859	6.992	1.074	0.924	0.401	0.988	0.030	8.066	1.312
Std. Dev.	0.089	1.274	0.578	0.918	0.353	0.148	0.180	1.416	1.023

Mean and standard deviation of nightly sleep feature values across 4,682,978 nights overall 33,152 individuals.

### Static sleep landscape reveals major sleep cluster and insomnia-like minor sleep clusters

To capture a persistent sleep phenotype, we constructed non-overlapping periods of 3–6 consecutive nights, which we refer to as sleep periods. We obtained 766,885 sleep periods after applying the exclusion criteria, and we extracted the mean and standard deviation of the 9 nightly indexes over each period. We performed Uniform Manifold Approximation and Projection (UMAP) with 2 components on our 766,885 by 18 feature matrix to obtain a 2-dimensional representation of the data (Fig. 1d).

Figure 1d shows the 2-dimensional representation as a scatter plot, where the first UMAP component is the x-coordinate and the second UMAP component is the *y*-coordinate for each point. We observed the data from 5 clusters that are substantially isolated from each other: one major cluster with four minor clusters off the left side. We used DBSCAN to identify the clusters, though the precise clustering method is flexible given the large space between clusters. For the major cluster (Cluster 0), we identified 650,339 periods (Fig. 1d, in blue). For the minor clusters, we identified 56,131 periods in Cluster 1 (Fig. 1d, red), 45,488 periods in Cluster 2 (Fig. 1d, green), 14,845 periods in Cluster 3 (Fig. 1d, yellow), and 82 periods in Cluster 4 (Fig. 1d, pink).

To understand whether each cluster represents a sleep phenotype, we examined the characteristics of the sleep periods that make up each cluster. Figure 1f shows a typical sleep period from each cluster. The subfigures show the long and short sleep windows from all nights of each sleep period. Short windows, shown as thick lines, are less than 3 h in length and long windows, shown as thin lines, are greater than 3 hours in length. As illustrated in Fig. 1f, nights can include one or more sleep windows, with the most common nightly combinations of long, short, long + short, long + long, and short + short. In each night with multiple windows (represented jointly by a ‘+’), a stretch of wakefulness >60 min in duration separates the two windows.

We found that despite compression through UMAP, each cluster could be described by a characteristic that dominated the cluster (Fig. 1f). Cluster 0 consisted of sleep periods where every night of the 3–6 nights had exactly one long window. The majority (84.8%) of all sleep periods are part of Cluster 0, while the remaining sleep periods (15.2%) are part of Clusters 1–4, or minor clusters. Cluster 1 consisted of sleep periods with at least one short window night, which describes nights where sleep is interrupted or very short. Cluster 2 consisted of sleep periods with at least one long+short window night. Cluster 3 consisted of sleep periods with at least one long + long window night. Cluster 4 consisted of sleep periods comprised only of nights with very short sleep. We believe that the interaction between the UMAP algorithm and the LW Count and SW Count features, which only take on a small number of possible values, played a major role in the existence of our cluster characteristics. We treated each cluster as a high-level sleep phenotype for our initial temporal analysis.

The minor clusters all exhibited some insomnia-like patterns, marked by short sleep duration or sleep disruption, as defined in the DSM-5-TR criteria^12^. DSM-5-TR describes middle insomnia as the inability to maintain sleep, defined by frequent waking during the night after sleep onset beyond 20–30 min, and late insomnia as early-morning wakefulness, defined by waking before sleep reaches 6.5 h. Clusters 1–4 exhibit nights either containing at least a 60-min block of wake or less than 6.5 h of total sleep. We describe these clusters as insomnia-like because, if these patterns continued for at least 3 months, an individual might be diagnosed with insomnia according to the DSM-5-TR insomnia disorder criteria.

### High-level sleep phenotype is dynamic within individuals over time

**Fig. 2:**
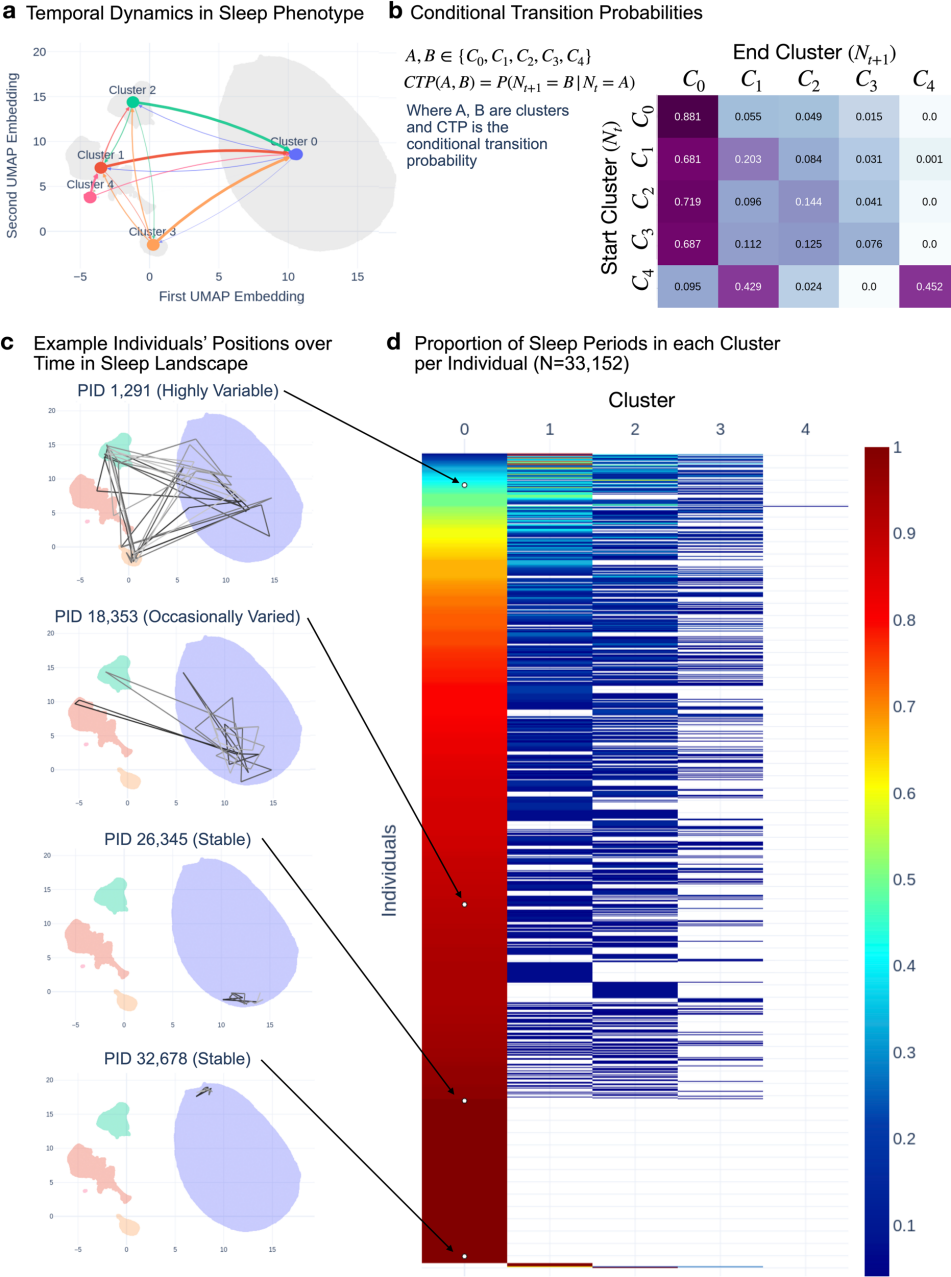
Temporal dynamics in sleep phenotypes. A graph model of sleep phenotypic change over time. **a** Illustrates graph approach to capture temporal changes in sleep across a population, using conditional transition probability between clusters. Arrows represent transitions, color indicates the initial cluster, and line thickness indicates conditional transition probability. **b** Adjacency matrix heatmap indicates conditional transition probability between pairs of clusters. Rows sum to one. **c** Examples of individuals with different variabilities in cluster. Note variable individuals transition between minor clusters, and stable individuals occupy different positions in major clusters. **d** Proportion of periods in each cluster across all individuals. Individuals are sorted by time spent in the major cluster. The blue, teal, and yellow colors at the top of the left column show the highly variable individuals who spent minimal time in the major cluster. The orange to red show the occasionally variable and stable individuals. White circles in the major cluster column indicate the position of the example individuals.

To test whether individuals’ sleep phenotype changes over time, we analyzed the distribution and transition patterns of the sleep periods for each individual (mean *n* = 23.13 ± 13.74 sleep periods for 33,152 individuals). First, we assessed whether an individual’s sleep periods tended to be evenly or unevenly distributed across clusters. A heavily uneven distribution, resembling a Dirac delta distribution, would indicate that an individual’s sleep phenotype is static over time (i.e., their sleep phenotypes were not evenly distributed across clusters but heavily localized to one cluster). A more even distribution would indicate more sleep phenotype changes over time. Figure 2d shows the within-individual distribution of sleep periods across clusters for every individual in our dataset. 71.5% of individuals spend >5% sleep periods in minor sleep clusters, indicating a partially uneven distribution. 9.2% of these individuals spend 40% or more of their total sleep periods in one of the minor sleep clusters, indicating a more even distribution. These are individuals who appeared to consistently move between sleep phenotypes.

Second, we tested whether transitions between temporally adjacent sleep periods from an individual indicate typical changes in sleep phenotype. We constructed a directed graph where each cluster is a node, and each edge weight is the number of transitions between two nodes divided by the number of transitions starting from the first node (Fig. 2a). In other words, the edge weight was the probability of transitioning to the end node given that one started in the start node (Fig. 2b). If sleep phenotype was static over time, the diagonal of the adjacency matrix in Fig. 2b would have been the strongest weight in each row. However, this was not the case, as the first column (Cluster 0) was the strongest in each row. This temporal transition model showed that the sleep phenotype was not stationary over time.

Transitions from Cluster 0 to the minor clusters occurred after 11.9% of Cluster 0 sleep periods. Transitions from the minor cluster to another minor cluster occurred after 28-31% of minor cluster sleep periods. Both results show that the stability of sleep phenotype varied across individuals and time.

### Subclusters describe fine-grained phenotypes; 1 recommended and 12 alternative phenotypes

Katori et al. discovered that subdivisions of each cluster allowed them to construct more distinct sleep phenotypes. Following this finding, we explored subdividing our clusters as well. Katori et al. used a density-based clustering method, which is not well suited for UMAP embedding representations because the UMAP algorithm does not preserve the density information of the original feature space. Thus, we applied a geometry- and a rule-based approach (see Section “Subcluster Construction in Methods”), obtaining 7 subclusters from Cluster 0 and 3 subclusters of Cluster 1.

We applied a rule-based subdivision for Cluster 1 after finding that Cluster 1 exhibited multiple sleep patterns. We found that a subset of sleep periods in Cluster 1 contained nights with multiple long sleep windows and extracted these sleep periods as Subcluster 1c. This is shown by the LW Count (the number of long windows per night on average in a sleep period) of Subcluster 1-c being greater than 1, as shown in Table 3 (LW Count, 1-c: 1.188). We extracted another set of sleep periods in the remainder of Cluster 1 that exhibited shorter than typical long windows (LW Len, 1-b: 4.606 h) as Subcluster 1-b. The remaining sleep periods are described as Subcluster 1-a.

**Fig. 3:**
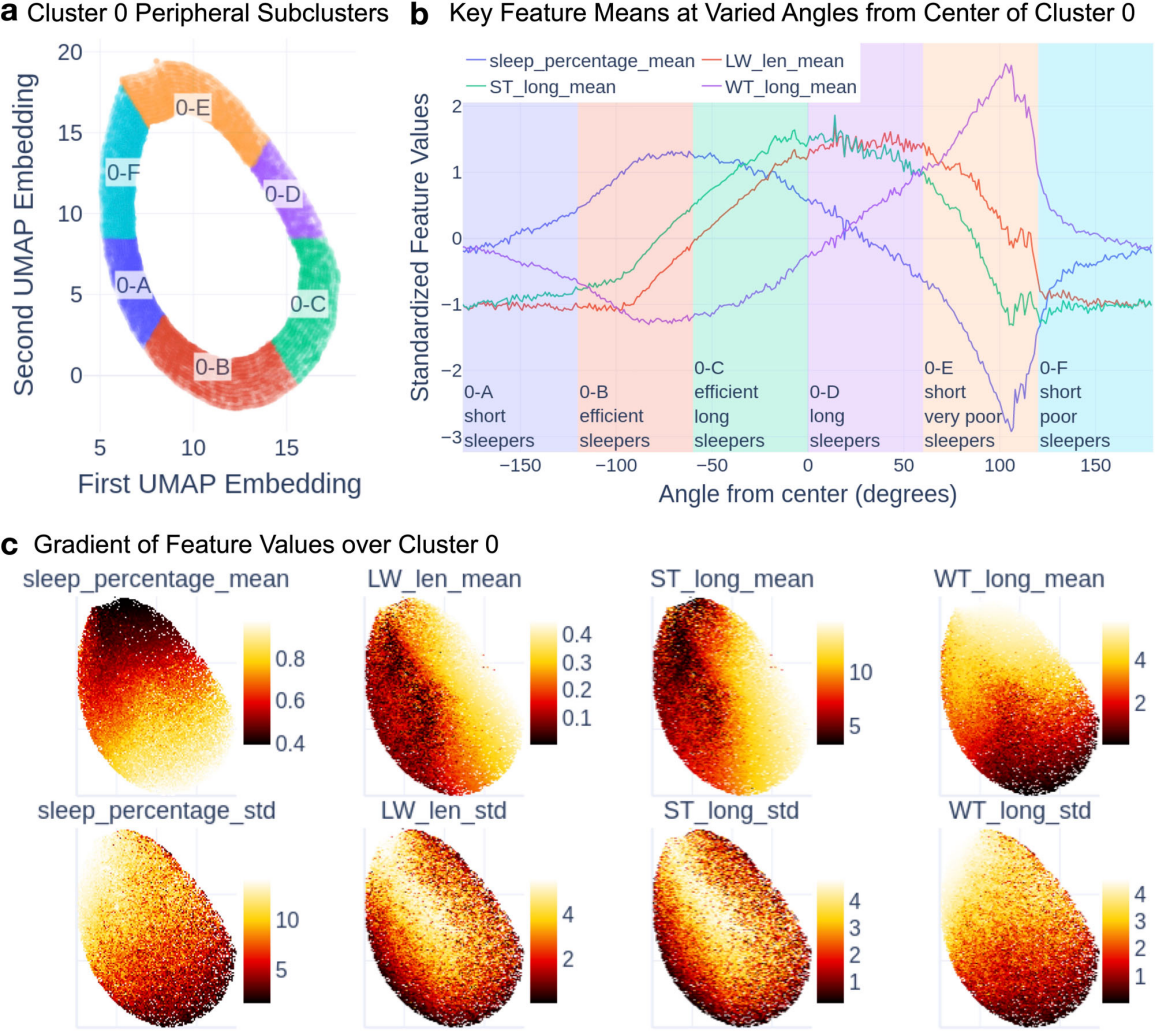
Enriching Sleep Phenotypes in Cluster 0. Here we describe how we subdivided clusters to obtain more fine-grained phenotypes. Rather than a single set of behaviors, Cluster 0 is best described as a gradient of different sleep patterns, where the periphery of the cluster describes more recognizable sleep patterns than the center. **a** We extracted the peripheral sleep periods and divided them into 6 subclusters based on their angle from the centroid of Cluster 0. **b** We show how key sleep features, standardized by the Cluster 0 mean and std, changed as we swept around the periphery of Cluster 0. The background colors on the right match the colors of the subclusters in (**a**). **c** We show the feature value gradients across Cluster 0 as heatmaps. The bottom row shows the standard deviation, while the top shows the mean.

Figure 3a illustrates how we geometrically subdivided Cluster 0. Feature values in Cluster 0 form a gradient (Fig. 3c), where the peripheral regions form recognizable sleep patterns. We extracted the peripheral region of Cluster 0 and divided it into 6 subclusters (60° regions of the periphery) based on their angle from the centroid of Cluster 0, as shown in Fig. 3a. We observed that key feature values show quasi-regular change by the angle from the center of Cluster 0, (Fig. 3b, starting at −180° and going counterclockwise). The smooth gradient around the periphery of the cluster and the cohesive character of each peripheral region suggest that other clustering solutions are possible, as no hard delineations are apparent in the underlying features. The center of Cluster 0 is the average of all peripheral regions (Table 3).

**Table 3:** Key features of alternative subclusters in the minor clusters

	Features: mean (S.D.) over subcluster					
Subcluster	LW count	SW count	Sleep percentage	LW length (h)	SW length (h)	*N* (sleep periods)
0 (rec.)	0.999 (0.014)	0.000 (0.000)	0.870 (0.045)	8.087 (0.842)	–	650,339
1-a	0.811 (0.064)	0.215 (0.101)	0.784 (0.073)	6.384 (1.034)	0.196 (0.233)	45,354
1-b	0.620 (0.096)	0.407 (0.180)	0.700 (0.105)	4.606 (1.184)	0.405 (0.422)	4937
1-c	1.145 (0.108)	0.236 (0.125)	0.769 (0.064)	7.412 (1.236)	0.411 (0.311)	5840
2	1.000 (0.002)	0.201 (0.080)	0.821 (0.053)	7.757 (1.045)	0.331 (0.212)	45,488
3	1.188 (0.055)	0.000 (0.000)	0.824 (0.051)	8.250 (0.978)	–	14,845
4	0.000 (0.000)	0.854 (0.191)	0.552 (0.167)	–	0.510 (0.286)	82

Key feature means of minor cluster alternative sleep phenotypes in comparison to recommended sleep phenotype.

We refer to the central Cluster 0 subcluster as the recommended sleep phenotype, as it most closely resembles the recommended 8-hour monophasic adult sleep phenotype. We refer to the peripheral Cluster 0 subclusters and minor clusters’ subclusters as the alternative sleep phenotypes, as they deviated from the recommended phenotype. Two of the Cluster 0 subclusters exhibited stronger signs of poor sleep with high mean wake times during long sleep windows (0-E: 1.192 h, 0-F: 1.858 h) and lower sleep percentage (0-E: 84.4%, 0-F: 78.9%) than the other Cluster 0 subclusters. We describe these two as insomnia-like for our analyses, though this interpretation is subjective, resulting in a total of eight insomnia-like phenotypes.

### All clusters typically transition to the recommended phenotype

**Fig. 4:**
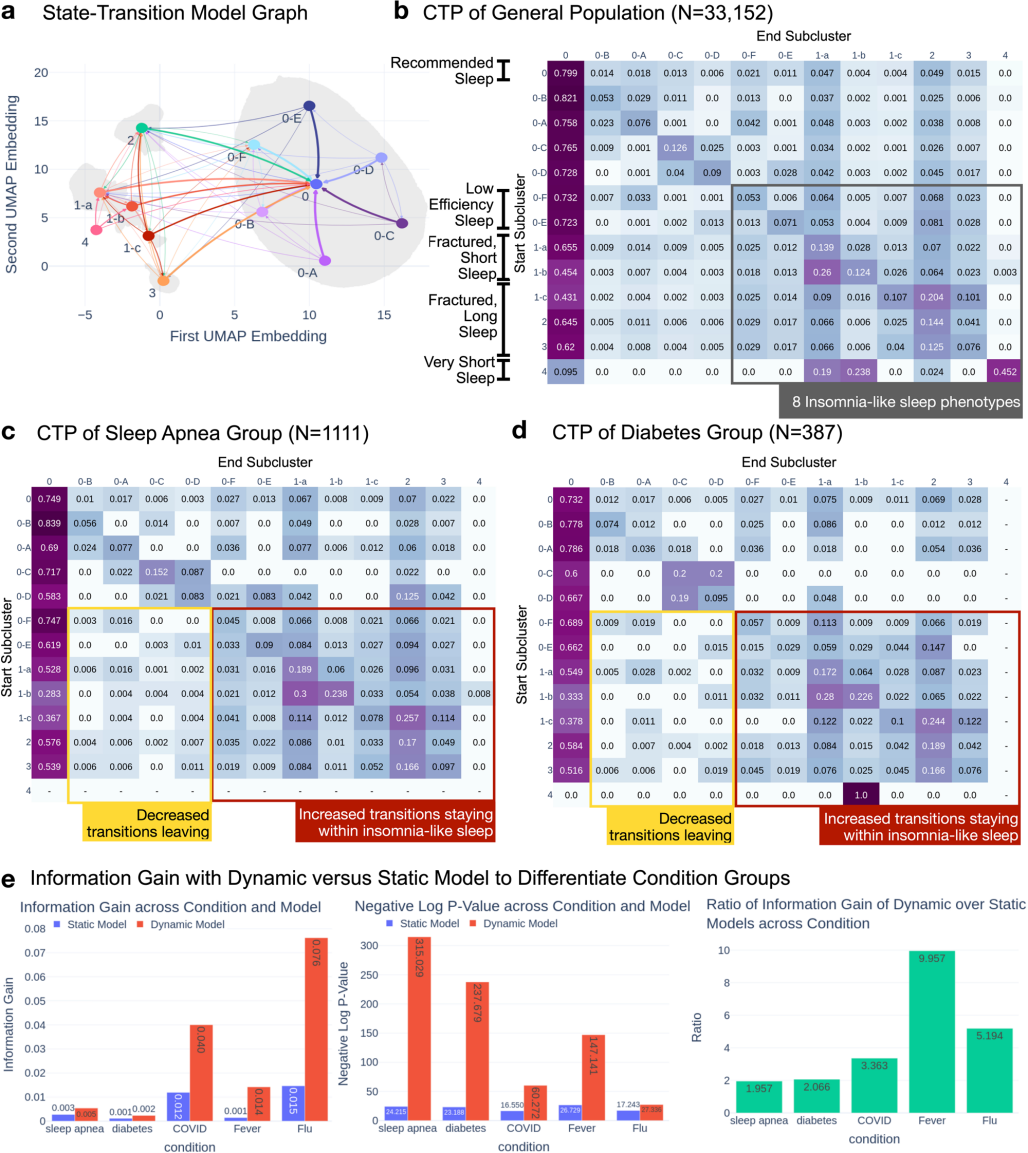
Assessing chronic and acute health conditions with temporal dynamics. Subcluster-derived graph of observed transition probabilities between and within clusters. **a** Directed graph model modified to assess transitions across all subclusters. **b**–**d** Subcluster transition matrices for all individuals (**b**), individuals with self-reported sleep apnea (**c**), and individuals with self-reported diabetes (**d**). **e** Comparative information gain in classifying conditions (left), associated *p*-values (center), and the relative ratio of red/blue (right), from transition matrices (red) vs. instantaneous location only (nodes without edges; blue).

With an enriched set of sleep phenotypes derived from subclustering, we reassessed the temporal dynamics of our population (Fig. 4a). We recalculated the conditional transition probabilities of the entire population using the 13 subclusters and revealed new heterogeneity (Fig. 4b). The clearest visual feature is the dark purple column on the far left. The column represents the likelihood of transitioning in the next sleep period to the recommended sleep phenotype from each other phenotype. The recommended phenotype transitions to itself with probability 0.799. The likelihood of transitioning to the recommended phenotype ranged from 0.431 to 0.821, excluding Cluster 4 (0.095). Alternative phenotypes in Cluster 0 were more likely to transition to the recommended phenotype (0.723–0.821) than those in the minor clusters (0.431–0.655). Remaining in or transitioning to the recommended phenotype was the dominant transition pattern.

### Lower sleep percentage phenotypes in Cluster 0 are more likely to transition to insomnia-like minor clusters

We examined whether lower sleep percentage phenotypes in Cluster 0 show differences in transition patterns from other phenotypes in Cluster 0. Of the Cluster 0 subclusters, those exhibiting lower sleep percentages (Subclusters 0-E and 0-F) are the least likely to transition to recommended sleep (probabilities of 0.723 and 0.732, respectively). On the other hand, Subclusters 0-A, 0-B, and 0-C, which were not considered insomnia-like, were the most likely to transition to the recommended phenotype (probabilities of 0.754, 0.765, 0.821, respectively). Similarly, Subclusters 0-E and 0-F were more likely than Subclusters 0-A, 0-B, and 0-C to transition to insomnia-like phenotypes in Subclusters 1-a (0.053-0.064 vs. 0.034-0.048) and 1-b (0.068-0.081 vs. 0.025-0.049). In summary, lower sleep percentage phenotypes in Cluster 0 transition to minor cluster phenotypes, which exhibit insomnia-like patterns more often than other Cluster 0 phenotypes. These findings suggest that sleep percentage in monophasic sleepers carries information on insomnia-like sleep interruptions in future nights.

### Insomnia-like phenotypes transition to subclusters with similar total sleep time over other insomnia-like phenotypes

We examined the transition probabilities of insomnia-like phenotypes to understand dynamics when insomnia-like individuals didn’t transition to recommended sleep (Cluster 0-center). Among the insomnia-like phenotypes, Clusters 1-a, 1-b, and 4 exhibits shorter total sleep times (6.58, 5.01, 0.55 h), while 1-c, 2, and 3 exhibits longer total sleep times (7.82, 8.08, 8.250 h). We refer to the first group as the short, fractured phenotypes and the second groups as the long, fractured phenotypes (Table 4).

**Table 4:** Means of key sleep features over Cluster 0 subclusters

	Key sleep features: mean (S.D.) over subcluster				
Subcluster	Sleep percentage mean	ST long mean (h)	WT long mean (h)	LW length mean (h)	*N* (sleep periods)
0 (center)	0.870 (0.045)	7.036 (0.795)	1.052 (0.389)	8.087 (0.842)	595,711
0-A	0.919 (0.017)	7.080 (0.407)	0.622 (0.135)	7.702 (0.406)	8800
0-B	0.876 (0.016)	6.546 (0.319)	0.933 (0.120)	7.479 (0.347)	10,855
0-C	0.921 (0.017)	8.319 (0.387)	0.718 (0.166)	9.037 (0.436)	6771
0-D	0.872 (0.022)	8.499 (0.514)	1.246 (0.204)	9.745 (0.484)	4270
0-E	0.844 (0.031)	6.373 (0.680)	1.192 (0.187)	7.565 (0.657)	11,621
0-F	0.789 (0.046)	6.987 (0.971)	1.858 (0.345)	8.845 (0.921)	12,311

Shows the key sleep features and number of sleep periods of the Cluster 0 subclusters.

The short, fractured phenotypes consistently transition to themselves over long-fractured subclusters. Subclusters 1-a, 1-b, and 4 transitioned to subclusters with short, fractured phenotypes at probabilities 0.167, 0.387, 0.948 respectively, while they transition to long fractured phenotypes at probabilities 0.044 (73.6% relative reduction), 0.113 (70.8% relative reduction), 0.024 (97.4% relative reduction) respectively. Conversely, the long, fractured phenotypes consistently transition to themselves over short-fractured phenotypes. Subclusters 1-c, 2, and 3 transition to subclusters with long, fractured phenotypes at probabilities 0.413, 0.210, 0.241, respectively, while they transition to subclusters with short-fractured phenotypes at probabilities 0.106 (74.3% relative reduction), 0.072 (65.7% relative reduction), 0.072 (70.1% relative reduction) respectively. Visually, these patterns manifest as darker squares of the adjacent similar subclusters (Fig. 4b). These findings suggest that when insomnia-like phenotypes do not transition to recommended sleep, they tend to transition to an insomnia-like phenotype with a similar total sleep time.

### Distribution of sleep phenotype shifts with diabetes, sleep apnea, flu, fever, and COVID-19

To assess the relevance of the static sleep landscape to health, we tested for differences between the general population and various abnormal populations (Fig. 4e). We tested whether the sleep phenotype categories based on our subclusters are relevant to various health conditions. For diabetes and sleep apnea conditions, we performed a chi-square test for independence between the frequency of sleep phenotype of the positive and negative groups for sleep apnea and diabetes as reported in the baseline self-report survey. We found that the positive groups were significantly different from the negative groups at *p*-values of 8.5 × 10^−11^ (sleep apnea; 10,017 neg vs. 1111 pos) and 3.0 × 10^−11^ (diabetes; 10,732 neg vs. 387) [chi-square test for independence between sleep periods from positive and negative individuals]. We compared the nights around a COVID-19 diagnosis, a flu report, and a fever symptom report to nights many weeks prior. We found that the frequency of sleep phenotypes was significantly different with *p*-values 6.5 × 10^−8^ (COVID-19; 147 individuals), 3.2 × 10^−8^ (flu; 2019 individuals), 2.5 × 10^−12^ (fever; 272 individuals) [chi-square test for independence within the individual between sleep periods from positive and negative time regions].

### Temporal dynamics contain 2–10× as much information about health conditions as static sleep phenotype

To assess the relevance of our temporal dynamics model to health, we tested for differences between the general population and various abnormal populations and compared the information gained between our dynamic model of sleep and the static sleep landscape model tested in the previous section. As shown in Fig. 4e, we tested whether the transitions based on our subclusters are relevant to various health conditions. We performed a chi-square test for independence between the transition probabilities of the positive and negative groups, as described in the previous section, of various health conditions, treating the frequency of transitions between subclusters as a categorical variable. We showed that, under this transition-based model, the positive groups were significantly different from the negative groups with *p*-values, 1.5 × 10^−137^ (sleep apnea; 10,017 neg vs. 1111 pos), 6.0 × 10^−104^ (diabetes; 10,732 neg vs. 387 pos), 6.7 × 10^−27^ (COVID-19; 147 individuals), 1.3 × 10^−12^ (flu; 2019 individuals), 1.2 × 10^−64^ (fever; 272 individuals) for sleep apnea, diabetes, COVID-19, flu, and fever respectively [chi-square tests for independence as described in prior section].

We compared the health relevance of this model to the static model using information theoretic metrics. We calculated the information gain of the static and dynamic models and found that the dynamic model yielded a several-fold increase in information over the static model; 1.97× in sleep apnea, 2.07× in diabetes, 3.36× in COVID-19, 5.19× in flu, and 9.96× in fever.

## Discussion

In this work, we found that sleep phenotypes are dynamic within individuals across time and that these dynamics contain information relevant to health conditions. We first replicated the methods proposed by Katori et al. to obtain a 2-dimensional embedding that contained phenotype-like clusters of 3–6-night sleep periods. We enriched our landscape by subdividing clusters to obtain 13 distinct sleep phenotypes. To study how populations and individuals move between phenotypes over time, we constructed a graph of within-individual transitions between sleep phenotypes over time, where subclusters were nodes, possible movements between subclusters were edges, and the probabilities of moving between a pair of subclusters were edge weights. This analysis of temporal dynamics revealed that an individual’s current sleep patterns can indicate in what ways those patterns are likely to change soon. One such pattern suggested that a low percentage of time in deep sleep indicates a higher chance of insomnia-like sleep disruption in the following sleep period. We hypothesized that transitions between phenotypes (edge weights in our temporal dynamics graph) might carry more information about an individual’s health than the current sleep phenotype alone. We found that the dynamic transition model indeed carried several times more information about cardiometabolic-, respiratory-, and sleep-related health factors. These findings suggest that capturing temporal dynamics in sleep phenotype could improve individual and population-level sleep screening for health.

Our sleep landscape produced similar features and insomnia-like subclusters as Katori et al. Subcluster 0-center represented the NIH-recommended 8-h monophasic sleep phenotype. Subclusters 0-A through 0-F represented alternative monophasic sleep phenotypes, with 0-E and 0-F exhibiting the lowest sleep percentages (84.4%, 78.9%). Clusters 1–4 represented fragmented (wake > 1 h) or very short sleep (<6.5 h). While prior work did not quantitatively test whether the phenotype is associated with health, we found that the static sleep phenotype model significantly separated populations of diabetes and sleep apnea as well as time periods of flu, fever, and COVID-19. These initial results support the reproducibility of sleep phenotype landscapes and validate the relevance of such landscapes to sleep disorders.

We then investigated whether an individual’s sleep phenotype is stable over time. A period of 8-hour monophasic sleep is followed by a similar period 79.9% of the time. Alternative sleep phenotypes tend not to be stable (Subclusters 0-A–0-F: 5.3–12.6%, Clusters 1–3: 10.7–14.4%, Cluster 4: 45.2%), and instead transition to the recommended phenotype 43.1–82.1% of the time (excluding Cluster 4 at 9.5%). These findings suggest that sleep phenotype is not stable over time. As transitions to alternate sleep phenotypes were both spread across the population but also disproportionate in a small set of individuals, specific rates and transition patterns are likely population and study dependent.

Given that sleep, phenotype is not static and that 8 out of our 13 phenotypes are marked by some degree of insomnia-like patterns (short or disrupted sleep defined by DSM-5-TR), we hypothesized that an individual’s particular phenotype might suggest how likely they are to transition to an insomnia-like phenotype. Periods of relatively low sleep percentage, monophasic sleep were more likely to transition to disrupted or short sleep (22.6–25.9%) than higher sleep percentage monophasic sleep periods (7.4–15.1%). These findings suggest that low sleep percentage in monophasic sleepers may indicate susceptibility to insomnia-like sleep interruptions in future nights. Furthermore, when we group insomnia-like phenotypes by total sleep time, insomnia-like phenotypes transitioned to themselves 16.7–41.3% of the time. This further illustrates how insomnia-like sleep predisposes one to insomnia-like interruptions in future nights of sleep.

We further show that these temporal dynamics are relevant to cardiometabolic and respiratory health, along with sleep quality. We showed that diabetic and sleep apnea individuals are more likely to stay in insomnia-like sleep clusters (see Fig. 4c, d). We also showed that there are significant differences in distribution between the months prior to and the weeks around COVID-19, flu, and fever diagnoses (see Fig. 4e). Across conditions, we found a 2–10× information gain from a static to dynamic model of sleep. These results suggest that temporal sleep dynamics may be an informative feature in health screening tools.

At the individual level, our results suggest that temporal sleep dynamic features could improve algorithms detecting the presence of and tracking the severity of chronic health conditions, though future work is needed to assess this possibility. Further, researchers have assessed the potential for anomaly detection of acute illness (e.g., COVID-19 and flu) using sleep and physiological features^10,13,14^. Our work here suggests that modeling within-individual temporal dynamics may help account for substantial amounts of variability in different modalities used in algorithms. As a result, incorporating methods like those we describe here may improve algorithm performance both when identifying acute illness and potentially when screening for chronic conditions as well. Perhaps, especially for the latter, such approaches could support public health and population risk screening efforts in addition to the potential value for algorithms aimed at individuals. Significant work is needed to test these hypotheses in different conditions and populations.

The work we present successfully supports hypotheses of the value of dynamic features in longitudinal data, and it is intended to provide examples demonstrating the potential gains from approaches using such features. We caution against overinterpreting specific numbers in our findings, as our work has several limitations. First, health conditions were determined by individual self-report, and thus, some “healthy” periods may have contained unreported conditions. Second, the sample size for certain conditions was relatively much smaller than the general population they were compared against (e.g., Diabetes vs. General population: 387 vs. 33,152), and thus, the sampled condition population may not be representative of the entire condition population. Third, there are likely confounding factors beyond those we made any attempt to assess, and this could be the case at all stages of analysis. For example, at the sensor level, we cannot differentiate between an individual removing their ring and continuing to sleep and them removing their ring as they wake up. Fourth, the study only enrolled those who already owned an Oura Ring, which may be associated with socioeconomic biases in the population. Finally, our data was collected in 2020, during which the COVID-19 pandemic heavily affected the world, and so changes that occurred due to lockdown and heightened stress likely affect our analyses, particularly our general population statistics.

Future work may include features specific to outcomes of interest. For example, encoding the daily phase of sleep onset as a feature could improve our method’s ability to diversify phenotypes related to social jet lag and shift work. While we observed some patterns on the time scale of months, future work may study whether seasonal and monthly timescales are robust and informative dynamic patterns in their own right. Finally, because our aim was to perform an initial exploration of how sleep could be dynamic over time, many parameters that we used for feature extraction, clustering, and modeling could be optimized for those interested in particular health outcomes in future work.

Our work confirms that dynamical models and topological data analysis capture new information from sleep by viewing an individual’s sleep phenotype as a state they are in temporarily rather than as a persistent trait of that individual. More complex sleep models based on multimodal data will likely yield yet more information, fueling algorithms for short and long-term health prediction.

## Methods

### TemPredict study data collection

Our data was collected through the TemPredict study at the University of California, San Francisco, which was conducted in collaboration with Oura Health Oy. The University of California San Francisco (UCSF) Institutional Review Board (IRB, IRB# 20-30408) and the U.S. Department of Defense (DOD) Human Research Protections Office (HRPO, HRPO# E01877.1a) approved all study activities. All research was performed in accordance with relevant guidelines and regulations and the Declaration of Helsinki. Informed consent was obtained from all participants. We collected Oura Ring Gen2 data from 63,153 participants from January to December of 2020. Further details are contained in Mason et al. A baseline self-report survey collected comorbidity information, and daily self-report surveys collected symptom reports as well as COVID-19 test data. Sleep stages (4-stage: light NREM sleep, deep NREM sleep, REM sleep, and wake) at 30-s granularity and sleep/wake onset (start and end times of a series of contiguous sleep stage predictions) were obtained for each night of sleep from the Oura Ring. The Oura Ring predicts the sleep stage with a proprietary algorithm that has been validated against Polysomnography (PSG) and Electro-encephalogram (EEG) by Miller et al., Ghorbani et al., and Zambotti et al.^15,16,17^. PSG-validated sleep stage data were available for 4,682,978 nights across *n* = 33,152 participants from January to October 2020. We describe the predicted sleep stages as part of the sleep summary feature set, which also includes predicted wake and sleep onset. We did not directly perform power analysis. The previous publication on sleep clustering (Katori et al.) was the largest (~100 K nights); because we had ~5 M nights (~50×), we used all the data available to discover insights instead of conscribing analyses to a large enough sample to ensure the significance of any one potential effect.

### Adapted data preprocessing and feature extraction

Our data preprocessing consisted of two stages. The first stage involved filtering individuals, cleaning outputs from the classification algorithm, constructing sleep-wake periods, and extracting the common sleep features. The second stage involved selecting sleep periods (3–6 nights) and then constructing our 18 sleep features from the selected sleep periods. Our preprocessing and feature extraction steps closely followed Katori et al. because our aim was to recapitulate the feature distributions their work observed.

In the first stage of data preprocessing, we handled the data at a nightly scale. First, we filtered individuals who did not have any nights with sleep summary features. Next, we removed nights with missing or only awake predictions from the sleep staging algorithm. We used the predicted sleep onset and wake onset to filter out predicted nights that were less than 30 min and occurred in the same day (determined by beginning within 12 h before or after midnight) as a longer predicted night. While these data may represent short naps, a majority of these sleep predictions overlapped with or occurred during the respective longer predicted night, suggesting that these sleep predictions were more likely artifactual and thus should be excluded from analysis. We removed nights with missing, faulty, or questionable data because the featurization of such nights misrepresents peoples’ sleep behavior.

Next, we constructed sleep windows from nights of sleep (determined previously by predicted sleep and wake onset). We treated the highest level of the sleep stage predictions as wake and the other three levels as sleep and then followed the same steps to construct sleep windows as Katori et al. This consisted of four steps: (a) changing contiguous wake predictions less than 10 min to sleep, (b) changing contiguous sleep predictions less than 10 min to wake, (c) connecting contiguous sleep predictions that were separated from other sleep prediction segments by 60 min of contiguous wake predictions as a single sleep window, and (d) making sleep windows less than 3 h into short windows and the remaining long windows. Further detail and a figure showing these steps can be found in the Katori et al. manuscript. Steps (a) and (b) smooth out sudden changes (e.g., 30 s of wake in a 6-h sleep window) that are likely the result of noise in the sleep stage predictions. Steps (c) and (d) encode breaks in sleep and differences between long and short sleep windows as features.

Finally, we constructed the nightly sleep features following the steps and naming conventions of Katori et al. For each sleep window, we calculated the wake time and sleep time during the window based on the sleep-wake predictions prior to the sleep window construction steps (before step A from the previous paragraph). These are the WT Long and ST Long features for Wake Time and Sleep Time in a long window (WT Short and ST Short for short windows). These features encode the amount of time spent asleep and the amount of time spent awake during a sleep window. We also calculated the total length of the sleep window and called this feature LW Length or SW Length for Long Window length or Short Window length. All these features are measured in time (hours). These features are typically extracted from sleep-wake time series to analyze sleep disorders. We also calculated the number of sleep windows of each type in a night as a feature, with LW Count and SW Count representing Long Window Count and Short Window Count. Multiple short windows often occur on nights with fractured sleep. We interpreted these features to indirectly represent the number of disruptions (>60 min of wake) in a night. Finally, we constructed Sleep Percentage, which represents sleep efficiency and was calculated as the total sleep time across all long and short windows divided by the duration of the sleep period, from sleep onset to wake onset. This provided 9 nightly sleep features. We chose these features because Katori et al. had shown that these features effectively diversify a variety of observable sleep phenotypes.

In the second stage of data preprocessing, we constructed a set of candidate sleep periods and filtered sleep periods based on our exclusion criteria and extracted our sleep features from the filtered sleep periods. Candidate sleep periods were extracted in the form of non-overlapping groups of 6 consecutive nights from an individual. Candidate sleep periods contained varying quantities of missing data. So, we filtered the candidate sleep periods by exclusion criteria to maintain data quality. Sleep periods were required to have <5 hours of non-wear time for each contiguous 3-night section of the 6 nights as well as ≥3 nights of data. Erroneous sleep periods of nights with no sleep windows were removed as well. These filtered and cleaned sleep periods could have between 3 and 6 nights of data. The parameters for these criteria followed our research hypothesis that peoples’ sleep behavior (as measured by our features) is not static over time. Sleep periods less than 3 days would be substantially affected by outlier nights and thus not allow for meaningful mean and standard deviations of features. Sleep periods greater than a week might include substantial behavioral changes, which could prevent an analysis of how sleep behavior changes over time. Thus, we chose a similar number of days to prior work and found that this was sufficient to test our research hypotheses. This resulted in 766,885 sleep periods to be used for further analyses. Finally, we calculated the mean and standard deviation of the zero-filled nightly sleep features over however many nights were in each sleep period, creating the 18 sleep period features we used in our analysis.

### Adapted dimensionality reduction and clustering

Like Katori et al., after constructing our feature set of sleep periods, we performed dimensionality reduction using Uniform Manifold Approximation and Projection (UMAP) to construct a 2-D sleep landscape from the first two UMAP components. UMAP is a non-linear dimension reduction algorithm that seeks to learn the manifold structure of data and find a low-dimensional embedding that preserves the essential topological structure of that manifold. We performed UMAP on the standardized, zero-filled sleep period features with 15 neighbors, min distance of 0.1, 2 components, and the remaining default parameters from the UMAP python library. These UMAP parameter settings were chosen based on the characteristics of the dataset (size and closeness of samples) as suggested on the UMAP documentation website. We performed Density-Based Spatial Clustering of Applications with Noise (DBSCAN) to obtain 5 distinct clusters (the number of clusters inferred by DBSCAN from data) that resemble the large clusters discovered by Katori et al. We validate the clusters by silhouette score and report the scores and cluster sizes in Table 5. By analyzing the average mean and standard deviation in feature values per cluster, we identified characteristics common to each cluster.

**Table 5:** Descriptors of major and minor clusters

	Cluster 0	Cluster 1	Cluster 2	Cluster 3	Cluster 4
***N*** **(sleep periods)**	650,339	56,131	45,488	14,845	82
**Silhouette score**	0.371	0.446	0.663	0.765	0.927

Numerical descriptors of major and minor clusters. A number of sleep periods and cluster validation statistic (mean silhouette score overall sleep periods in each cluster).

### Subcluster construction: rule- and geometric-based approaches

We believe Katori et al.’s choice to repeat DBSCAN on each initial cluster to discover subclusters could be improved upon in the context of our dataset. UMAP is not designed to preserve the local density information of the data. Due to UMAP’s design, a density-based clustering algorithm such as DBSCAN may not be well suited to identify sub-clusters, though it is effective for obtaining the more distinct, high-level clusters. However, like Katori et al., we hypothesized that there was nonrandom variance within some clusters that was associated with differences in the underlying sleep behavior, so we applied our own sub-cluster approach to test this.

Alternatively, to perform DBSCAN on each cluster, we applied two different approaches to constructing subclusters from a particular sleep cluster: a rule-based approach and a geometry-based approach. Our methods were based on the observation that each cluster exhibited a different characteristic in the variance of sleep phenotypes. Some clusters had a distinct characteristic that likely drives sleep periods’ membership in the cluster, whereas other clusters exhibit a smooth gradient of sleep parameters. The rule-based approach for identifying subclusters consists of attempting to identify a feature-based rule that lends to an intuitive understanding of the group and subdividing the group using the feature. The geometry-based approach applies a set of geometric rules to create regions that capture the gradient of feature values within a cluster to some degree. We designed these regions after inspecting visualizations of the sleep period features across the cluster. We tested whether a subcluster captured nonrandom variance by comparing the mean and standard deviation of relevant features to those of the cluster it belongs to.

The rule-based approach was applied to clusters that exhibited a distinct identity during preliminary analysis. Clusters 2, 3, and 4 all showed that a logical rule constructed from a few features would capture every sample in the cluster. For example, for Cluster 2, every period has exactly six long windows and one short window. Intuitively, this suggests that exactly one night had both a long and short sleep window and thus had fractured sleep. These identities are illustrated in Fig. 1f.

However, Cluster 1 did not have a clear identity during preliminary analysis. We noticed that the LW Count was close to the population mean while the ST Long was much less than the population mean. Initially, one might assume this suggests that sleep periods from Cluster 1 tend to have shorter long windows. However, we noted this effect can also be caused by a bimodal distribution of long window counts. We constructed a rule (No. of LWs in any night >1) to separate what is now Subcluster 1c from the rest of Cluster 1 and observed a divergence in the other features as well, leading us to conclude that Cluster 1c exhibits a distinct identity. We further separated Subcluster 1b with a rule (No. of SWs in any night >1) that led to another distinct identity. We ended with three subclusters in Cluster 1. Clusters 2, 3, and 4 remained unchanged. Key feature values of Subclusters 1a, 1b, and 1c are shown in Table 3.

For Cluster 0, the rule-based approach did not allow us to construct distinct subclusters. While every period in Cluster 0 contained exactly one long window per night, different regions of the cluster exhibited different amounts of sleep time, wake time, and total length, as can be seen in Fig. 2c, some of which could be associated with poor sleep. Because we wanted to separate the various sleep patterns into their own subclusters, we experimented with several approaches to create subclusters and chose the geometry-based approach.

The geometry-based approach involves recognizing the gradient of different patterns across the cluster and designing a set of geometric rules to segment the cluster into separate regions. As shown in Fig. 2c, we observed that the peripheral regions of the cluster describe more distinct sleep patterns than the center. So, we extracted the periphery of the cluster and divided it into six subclusters (60-degree regions of the periphery starting at −180° and going counter-clockwise) based on the angle from the centroid of Cluster 0, whose position was calculated as the mean of the x and y coordinates of Cluster 0 sleep periods. We did not attempt any kind of optimization by specific parameter separation because we believe that would suggest false precision as to a “right” subcluster selection being critical to the testing of our hypothesis. Instead, we sought a more arbitrary division out of concern that over-specifying the subclusters might (1) become circular if optimized by separation of features and (2) might distract from the desired test, which is that any reasonable subdivision of the cluster should reveal statistical differences consistent with meaningfully nonrandom change within a given cluster. We settled on 6 because it is a commonly used division of a circle, it allowed relatively large and well-represented subclusters, and it allowed easy visualization of several differences without creating an overwhelming number of comparisons. We confirmed that each region represents a different sleep phenotype that gradually transitions into the next sleep phenotype based on the means of key features, as shown in Fig. 3b. We show the means and standard deviations of these regions alongside the original cluster in Table 4.

### Silhouette coefficient

We calculate the validation statistic, silhouette coefficient, to report the quality of the clusters that the DBSCAN algorithm obtained. We used the sklearn.metrics.silhouette_score python function to calculate the statistic. This statistic first calculates the mean intra-cluster distance, $a$, and the mean nearest-cluster distance, $b$, for each sample, and the calculates the ratio of the difference to the max of these two quantities.

1 $${\rm{sillhouette}}\,{{\_}}{\rm{score}}=(b-a)/{\rm{max}} (a,b)$$

We report the mean silhouette scores of all points in each cluster in Table 5.

### Graph model of temporal dynamics

We modeled change in sleep over time with our temporal dynamics model. We aimed to capture the probability that an individual starting in a particular cluster or subcluster would transition to another cluster or subcluster. To do this, we first gathered every pair of consecutive sleep periods from the same individual (*N* = 699,552 transition pairs). Then, we constructed a directed graph where each cluster or subcluster was a node. Each directed edge represents a possible transition between consecutive sleep periods. We designed the edge weights to capture how likely an individual was to end in a particular node given the node in which they started. The edge weight was calculated as the conditional transition probability (CTP) as defined below.

We calculated conditional transition probabilities as follows. First, for each node, $A$, we counted the total number or transition pairs beginning at that node. Then, for each node, $B$, we counted the number of transition pairs ending at the node. We divided the number of transition pairs from $A$ to $B$ by the total number or transition pairs beginning at $A$ to calculate the conditional transition probability from $A$ to $B$. This calculation is shown in Eq. (2). The conditional transition probability was then assigned as the edge weight for the directed edge from node $A$ to node $B$. We repeated this for every possible ordered pair of nodes and assigned edge weights to every edge in our graph.

2 $${\rm{CTP}}(A,\,B)=P({N}_{t+1}\,=B\,{{|}}\,{N}_{t}=A)=\,\frac{P({N}_{t+1}\,=B\,\bigcap \,{N}_{t}=A)}{P({N}_{t}=A)}$$

The CTP’s from each node can be viewed as a probability distribution over the most likely next node, not only giving us context on which transitions are more and less likely in the population, but also giving us a method for identifying the relative likelihood of various acute and chronic conditions based on an individual’s transition patterns.

We used this transition model because it allowed us to understand trends across large populations of individuals. Longitudinal patterns of sleep across time have not been deeply studied on large populations at high resolution, partially due to the high dimensionality of longitudinal sleep time series, and as well as the challenge of collecting such data without wearable devices. However, our transition model captures reoccurring movement patterns within and across individuals, giving insight into population-scale trends. This allowed us to compare the strength of a particular trend amongst various subpopulations and temporal segments, and thus quantify how a particular dynamic characteristic might relate to sleep differences between these subpopulations.

### Selection of health conditions cohorts

To test the relevance of temporal dynamics to human health, we constructed various health condition cohorts. We selected two chronic conditions and three acute conditions to study. For the chronic conditions, we used entries from the baseline self-report survey to construct our cohorts. For the sleep apnea condition, we received 10,017 negative reports, 1111 positive reports, and 21,267 no-answer reports. For the diabetes condition, we received 10,732 negative reports, 387 positive reports, and 21,276 no-answer reports. No-answer individuals were excluded from each respective analysis.

For the acute conditions, information was obtained from a daily symptom survey delivered on participants’ mobile devices (typically phones). We compared the 14 days before and 14 days after the date that the condition was reported with all data prior to 50 days before the report. Sleep periods were included if the start date of the sleep period was within the selected time range. These time ranges were chosen so that the acute conditions were likely to be present in the region around the report and likely not be present in the region prior to the report, thus allowing us to test the relevance of temporal dynamics to the acute condition. The COVID-19 cohort contained 147 individuals. The fever cohort contained 272 individuals. The flu cohort contains 2019 individuals. In the 50 days before the report, individuals did not report any other acute condition. It should be noted that for these conditions, a multitude of time-varying events that could affect sleep, such as women’s cycling, changes in mental health, and behavioral events, were not taken into account.

### Comparing static and dynamic models of sleep: Pearson’s chi-square test and information gain

To understand the dynamics’ information relevance to health, we compared static models of sleep to dynamic models of sleep. To perform these comparisons, we (1) tested for the difference in distribution between the positive and negative cohorts of each distribution and (2) assessed the difference in information gain provided by one model over the other. In our dynamic model, we treated the transitions from each node to every other node, P, as one variable and the transition categories, C, as the other variable. The static model contingency table was constructed with the presence of the condition, P, as one variable and the static categories, S, as the other variable. These tables were used to perform statistical tests and calculate information gain (see Information Gain below). To simplify notation, we show our formulas using the dynamic model as one variable and the transition categories, C, as the other variable.

We performed the Pearson’s chi-square test^18^ to test whether the positive group is significantly different in distribution from the negative group for each condition. We compare the *p*-values obtained using the static versus the dynamic models. Pearson’s chi-square statistic was calculated as follows in Eq. (3), with O observed and E expected events.

3 $${{\rm{{\rm X}}}}^{2}=\sum \frac{{(O-E)}^{2}}{E}$$

It was performed using the scipy.stats.chisquare function in the SciPy Python library, with the positive group as the observed distribution, the negative group as the expected distribution, and the degrees of freedom set to be the number of categories for each respective model minus one.

Information gain is calculated by taking the difference between the entropy of the presence of condition variable, $H[P]$ and the conditional entropy of the presence variable conditioned on the respective model’s categorical variable, $H[{P\; |\; C}]$. We calculate information gain with Eq. (4).

4 $${IG}\left(P,C\right)=H\left[P\right]-H\left[P\,|\,C\right]$$

We take the ratio, ${IG}(P,{C})/{IG}(P,{S})$, to quantify the change in information gain between the static and dynamic models. to quantify the change in information gain between the static and dynamic models.

Our intention was to use information gain as a measure of effect size. A metric like Cramer’s V would simply measure the difference in mean values of a given comparison, whereas the metric of information gain reflects the shift in distribution separability, which is an important effect if methods like those we illustrate here will be used to support real-world algorithms. For example, information gain is often used to decide which features will be used for a split in a decision tree algorithm because it reflects the importance of a variable to separating the dataset by a given label. As such, we believe it is the most appropriate measure of effect size to compare the static and dynamic models.

## Data Availability

The data that support the findings of this study are available from Oura Health Oy but their data use policy does not permit us to make the data available to third parties without approval, and so are not publicly available. Therefore, those seeking to reproduce our findings should contact Ashley Mason, PhD, and Benjamin Smarr, PhD for an online application to access the study data portal. This application process will require requesters to make a written commitment expressing agreements to not duplicate data, to not share data with third parties, and/or other confidentiality precautions.

## Code availability

The underlying code for this study is not publicly available but may be made available to qualified researchers on reasonable request from the corresponding author.
